# Cognitive Bias for Learning Speech Sounds From a Continuous Signal Space Seems Nonlinguistic

**DOI:** 10.1177/2041669515593019

**Published:** 2015-10-18

**Authors:** Sabine van der Ham, Bart de Boer

**Affiliations:** Artificial Intelligence Lab, Vrije Universiteit Brussel, Belgium

**Keywords:** learning biases, frequency learning, continuous signal space, perception bias, evolution of speech

## Abstract

When learning language, humans have a tendency to produce more extreme distributions of speech sounds than those observed most frequently: In rapid, casual speech, vowel sounds are centralized, yet cross-linguistically, peripheral vowels occur almost universally. We investigate whether adults’ generalization behavior reveals selective pressure for communication when they learn skewed distributions of speech-like sounds from a continuous signal space. The domain-specific hypothesis predicts that the emergence of sound categories is driven by a cognitive bias to make these categories maximally distinct, resulting in more skewed distributions in participants’ reproductions. However, our participants showed more centered distributions, which goes against this hypothesis, indicating that there are no strong innate linguistic biases that affect learning these speech-like sounds. The centralization behavior can be explained by a lack of communicative pressure to maintain categories.

## Background

A central issue in speech evolution research is which aspects of human cognition have evolved specifically for speech or, rather, which aspects of cognition have undergone selective pressure related to speech (for a brief review, see de Boer, forthcoming). Examples of physical adaptations related to speech, though still controversial, are the evolution of the vocal tract (see de Boer & Fitch, 2010 for discussion) and voluntary respiratory control (MacLarnon & Hewitt, 2004). An example of a cognitive adaptation for speech is the ability to imitate vocalizations, including those of other species (Ackermann, Hage, & Ziegler, 2014).

The current research investigates the cognitive mechanisms involved in the chain of perceiving, learning, and reproducing a skewed distribution of speech-like signals from a continuous signal space. Although, perception, learning, and production may depend on different mechanisms, in a culturally transmitted system such as language, a cognitive bias at any level of this chain can have a nontrivial impact on the language as a whole. Specifically, a weak individual bias to generalize learned input may have a strong effect on how the language is passed on to successive generations (Kirby, Dowman, & Griffiths, 2007). To get a complete understanding of the impact of auditory biases on language, we must first understand the generalization mechanisms that are involved in reproducing learned speech-like sounds.

Parts of the perception–learning–reproduction chain have already been investigated. The distributional learning mechanisms involved in language learning have been explored thoroughly by psycholinguists (for a comprehensive review, see Lany & Saffran, 2013). However, typical psycholinguistic distributional learning methods only look at passive knowledge (for instance, by measuring looking times or sucking patterns on special pacifiers) but do not look directly at reproduction of the learned distributions. We are interested in what participants’ direct reproductions reveal about the generalization behavior of the learner.

A method that does include the entire chain of perception, learning, and reproduction is the iterated learning (IL) paradigm (e.g., Kirby, Cornish, & Smith, 2008). In a typical IL experiment, the input stimuli for a participant are the reproductions of the previous participant, thus mimicking cultural evolution. The input stimuli can be anything from an artificial slide whistle language (Verhoef, 2012) to drawings (Garrod, Fay, Lee, Oberlander, & MacLeod, 2007; Tamariz & Kirby, 2015). These experiments show that the process of cultural evolution amplifies small biases, resulting in increased structure and learnability of the transmitted systems of signals. However, these experiments mostly give a qualitative insight in the learning mechanisms involved. While our experimental design was largely inspired by the IL paradigm, we focus on a simpler individual learning task.

Ferdinand, Thompson, Kirby, and Smith (2013) is a great example of such a basic, straightforward learning task. They investigate a frequency learning task in which adults learn the distribution of colored marbles across different containers and later reproduce these distributions from memory. They found that the strategy adults use when processing frequency information depends on the cognitive load of the task: If it was easy (one container with 10 marbles in two different colors), participants reproduced distributions exactly; when it was difficult (six containers with ten marbles in two colors), they overgeneralized the most frequent color. Ferdinand et al.’s (2013) research is closely related to our research but focuses on distribution of discrete information (colors of marbles), whereas we focus on continuous information (the pitch of a speech-like sound).

### Research Question, Hypotheses, and Predictions

The current research investigates whether humans show a pattern that reveals selective pressure for communication when learning skewed distributions of speech-like sounds from a continuous signal space. Due to the high cognitive load, we expect that participants’ reproductions will reveal generalization behavior; it is impossible to exactly remember and match 12 distinct items with minimal training. However, the generalization behavior can go two ways: either with a peak shift toward the center of the signal space or toward the periphery.

Wedel’s (2012) model predicts a general bias toward the center of a bounded distribution that operates at all times, unless there is a pressure to maintain differences between categories. Liljencrants and Lindblom’s (1972) model predicts a tendency to produce more peripheral sounds than those most frequently observed, as maximizing the distinctiveness of sound categories is beneficial in communication (see also, Chirkova & Gong, 2014).

As participants learn and produce speech-like signals, we expect that the production distributions are shifted toward the periphery of the continuum in order to maximize their distinctiveness. Observing a more skewed reproduction of continuous signals would be indicative of a learning mechanism that is somehow specialized for language. Alternatively, if there is no significant shift of the peak, this may indicate that participants use a strategy similar to probability matching (e.g., Ferdinand et al., 2013), though the kurtosis of the reproduced distribution may still be different.

### The Experiment

**Figure 1.**
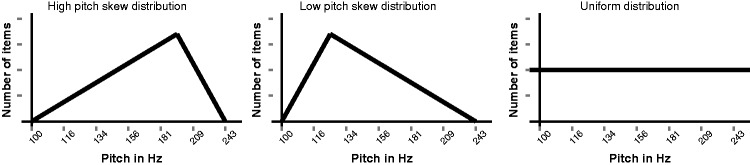
The training distributions in the two skewed conditions and the uniform distribution conditions, and which served as a control.

We ran a frequency learning experiment (presented as an alien language course), in which participants learned sounds coming from a continuous signal space. The training stimuli were manipulated so that the underlying distribution was skewed. Depending on the condition they were assigned, participants were exposed to a set of stimuli that was skewed to the lower pitch range or to the higher pitch range, or received a training set that was uniform (see Figure 1). We recorded the training stimuli, participants’ direct imitations, and their reproductions from memory. The latter two were created by using acoustic signals generated with a graphical computer interface; participants never used their voice (see Methods section for more details).

## Results

We investigated the difference between the distribution of the training stimuli and the distribution of the participants’ reproductions. For each combination of round, condition, and participant, the difference between the position of peaks in the input and the output was calculated. For each participant, the difference between the positions of the peaks was then used as a data point for a Wilcoxon’s rank-sum test to test whether the median of the shifts of all participants was different from zero.

**Figure 2.**
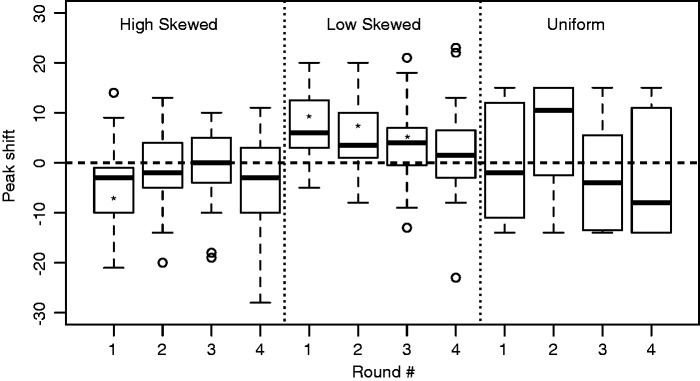
Boxplots that show the fitted peak of the output distribution compared to the peak of the distribution of training stimuli (at 0). The uniform input distribution is included to show that there are no extreme condition-independent biases toward high or low pitch. The peak shift values represent the number of steps in the signal space which was discretized into 30 sounds. See Materials and Stimuli section for details.

For all participants, the position of the peak of the reproduced distributions was estimated by fitting a triangular distribution, spanning the original input range, with the highest likelihood to the observed input and output. This was done for all rounds in the two skewed conditions, but not the uniform distribution as the Maximum Likelihood estimation assumes a triangular distribution. The uniform condition was added to ensure that there was no bias for high- or low-pitched sounds (see Figure 2).

Overall, participants produced an output that was different from the input data, but not toward the periphery of the distribution, as the domain-specific hypothesis predicted. In the condition where signals were skewed to low-pitch sounds, there was a significant overall shift toward the center: median shift size = 4, *N* = 20; *p* = .005, *V* = 180 with the Wilcoxon rank-sum test. In the high-pitch skew condition, the median also moved toward the center, but this overall shift was not significant: median shift size = −3, *N* = 21; *p* = .3832, *V* = 90.

We also looked at the behavior per round. For each round, the difference between the position of the peaks of the training and output data was measured for each participant and used as a data point for a Wilcoxon’s rank-sum test. Generally, we observe a decrease in the shift of the peaks, indicating that with each round, participants were increasingly better at matching their reproductions to the training stimuli. This was significant for all rounds but the last in the low-pitch skew condition and significant in the first round of the high-pitch skew condition. See Table 1 and Figure 2 for a clear overview of the statistics.

**Table 1.** Distance to the Median for Each Round in the Two Skewed Conditions.

High-pitch skew condition					Low-pitch skew condition				
Round no.	Distance to median	*p*-value	*V* stat.	*N*	Round no.	Distance to median	*p*-value	*V* stat.	*N*
1	−3	0.034	177	21	1	6	0.001	15	20
2	−2	0.676	128	21	2	3.5	0.005	13	18
3	0	0.936	97.5	21	3	4	0.042	50	20
4	−3	0.108	148.5	21	4	1.5	0.261	74.5	20
Overall	−3	0.382	90	21	Overall	4	0.005	180	20

*Note.* Due to technical failure during Round 2, the data of two participants were excluded in the low-skew pitch condition. The distance to median values represents the number of steps in the signal space which was discretized into 30 sounds and ranged from 100 to 243 Hz. See Materials and Stimuli section for details.

## Discussion

The present study investigates the biases involved in learning and reproducing sounds from a continuous signal space. We found that two biases appear to influence the behavior on the task. First, a tendency to centralize the distribution of the reproductions toward the center of the signal space, which is probably related to learning and generalization mechanisms. This behavior was strongest in the low-pitch condition. Second, behavior that is similar to probability matching, which was strongest in three rounds of the high-skew condition. Neither bias shows evidence of being specially adapted to a linguistic or communicative function. This is expected from a general-purpose learning strategy but not from a language-specific one. The latter would benefit from moving sounds to the periphery of the signal space to make them more distinctive. An explanation for the current results is that the signals may not have been speech-like enough, or the task was not sufficiently framed as a speech-learning task to elicit speech-learning mechanisms.

Other factors must play a role in learning speech. If only the observed general purpose learning mechanisms were used to learn speech signals, the result would be that those signals would come to sound increasingly similar, as the observed mechanisms tend to decrease the difference between the two different original input distributions. Such erosion of differences would eventually lead to collapse of distinctiveness and merging of different categories of sounds (see also the first experiment in Kirby et al., 2008, where a culturally transmitted lexicon becomes increasingly underspecified).

An important factor in the preservation of linguistic sound categories is the functional load of a sound distinction (Wedel, Kaplan, & Jackson, 2013). However, in the current experiment, there is no such (linguistic) factor as functional load: The only information participants have is the range of the continuum and the observed distribution. This appears not to be sufficient in itself. The choice to exclude functional load was made because we wanted to investigate whether the learning of a single asymmetrically distributed linguistic signal in itself would lead to the distribution becoming more asymmetric. Including functional load implies a contrast between at least two signals, and in such a task, even general learning mechanisms may lead to increased distinctiveness of signals. The question arises then whether this really was a linguistic task. While we aimed to design a task that should involve language learning strategies, participants generally reported that they did not experience it as though they were learning a language. If the centralization bias was driven by the idea that the task was nonlinguistic, then different behavior may be expected for a more linguistic task, and this can be tested experimentally.

When does a sound become a linguistic sound? Participants generally reported that they were missing “the point” of learning the alien language: The lack of meaning space and lack of communication reduced the linguistic value of the task. Manipulating the presence of a communicative component and meaning space in a follow-up experiment is expected to affect the learning strategy of the participants. Our approach to understand the impact of auditory biases in the evolution of speech is to first get a more thorough understanding in the mechanisms involved in all aspects of speech, including learning and reproduction. In future experiments, we will investigate this further by comparing biases involved in learning signals from a continuous signal space in different modalities.

## Methods

### Participants

In total, 63 participants with self-reported normal hearing participated in the study; 41 participants participated in the skewed conditions of the study (18 male, age range: 19–49, mean age: 26.2). The remaining 22 participants did the uniform condition (7 male, age range 18–32, mean age 23.1). Participants were partly recruited from the Berlin Summer School, but the majority was undergraduate student at the Free University Brussels. None of the participants was a native speaker of a tonal language. They received monetary compensation for their participation.

### Materials and Stimuli

The experiment was built in a java applet that was run from a laptop. The stimuli were taken from a continuous signal space that was an/a:/sound that varied only in pitch, ranging from 100 to 243 Hz. The signal space was discretized into 30 sound files; the difference between the sound files was always 3%. From this signal space, 12 sound files were pseudorandomly selected to be the training set that participants had to learn. Pseudorandomly because the underlying distribution of the training set was skewed: Depending on the condition, participants received more items in either the lower or higher frequency range. Depending on the condition, the peak of the distribution was set at either the first or third quarter of the continuum.

### Procedure

Participants were instructed that they were going to do the first lesson of an alien language class, which focused on a basic feature of alien languages: That is, aliens do not want to be boring and therefore prefer to say words in different pitch. A linguistic reason to differentiate was avoided. To become fluent in this alien language, it is important that participants are able to discriminate between pitch differences.

Participants were told that they were going to learn 12 items, and that they should not be worried if they fail to remember all 12 of them. In fact, as it is quite impossible to remember 12 novel items, they were told to find a way to try to remember as many items as possible. They were reassured that they would probably improve their performance throughout the experiment. After a practice round, in which participants were familiarized with the buttons and the procedure, they learned and reproduced four sets of 12 signals: variations of the same “alien,” mechanically sounding/a:/that differed only in pitch, ranging from 100 to 243 Hz.

Participants sat in front of a computer and wore headphones. They did four rounds which consisted of two phases: a Learning & Imitating phase and a Reproduction phase. During the Leaning & Imitating phase, participants went through the sounds one by one. They clicked on the LISTEN button on the screen to hear a sound that they had to imitate. The LISTEN button could only be pressed once, which was done to have the participants pay close attention. Then, to imitate the sound, they pressed the IMITATE button, which played a random reference sound taken from the signal space. So the pitch of this reference sound could be very different or very similar to the sound that they had to learn. Participants could then adjust the pitch of the reference sound by using the UP and DOWN arrows on the keyboard. Once they think they found the correct pitch to match the first sound, they could press SAVE to save their imitation and to continue to the next item.

In the reproduction phase, participants were asked to reproduce the 12 sounds that they learned. After pressing the button for the reference sound and adjusting the pitch, they could submit the item by pressing SAVE. After each round, participants received encouraging feedback to keep them going.
